# Significance of serum procalcitonin as biomarker for detection of bacterial peritonitis: a systematic review and meta-analysis

**DOI:** 10.1186/1471-2334-14-452

**Published:** 2014-08-22

**Authors:** Shi-kun Yang, Li Xiao, Hao Zhang, Xiao-xuan Xu, Pan-ai Song, Fu-you Liu, Lin Sun

**Affiliations:** Department of Nephrology, The Second Xiangya Hospital, Kidney Institute of Central South University, Changsha, Hunan 410011 China; Department of Nephrology, The Third Xiangya Hospital, Central South University, Changsha, Hunan Province 410013 China

**Keywords:** Procalcitonin, Diagnosis, Peritonitis, Meta-analysis

## Abstract

**Background:**

Bacterial peritonitis is serious disease and remains a diagnostic challenge for clinicians. Many studies have highlighted the potential usefulness of procalcitonin (PCT) for identification of bacterial peritonitis, however, the overall diagnostic value of PCT remains unclear. Therefore, we performed a meta-analysis to assess the accuracy of PCT for detection of bacterial peritonitis.

**Methods:**

We performed a systematic searched in MEDLINE, EMBASE, SCOPUS, China Biology Medicine Database (CBM), China National Knowledge Infrastructure Database (CNKI) and Cochrane databases for trials that evaluated the diagnostic role of PCT for bacterial peritonitis. Sensitivity, specificity and other measures of accuracy of PCT were pooled using bivariate random effects models.

**Results:**

Eighteen studies involving 1827 patients were included in the present meta-analysis. The pooled sensitivity and specificity of serum PCT for the diagnosis bacterial peritonitis were 0.83 (95% CI: 0.76–0.89) and 0.92 (95% CI: 0.87–0.96), respectively. The positive likelihood ratio was 11.06 (95% CI: 6.31–19.38), negative likelihood ratio was 0.18 (95% CI: 0.12–0.27) and diagnostic odds ratio (DOR) was 61.52 (95% CI: 27.58–137.21). The area under the receiver operating characteristic curve (AUROC) was 0.94. Use of a common PCT cut-off value could improve the DOR to 75.32 and the AUROC to 0.95. Analysis of the seven studies that measured serum C-reactive protein (CRP) indicated that PCT was more accurate than CRP for the diagnosis of bacterial peritonitis.

**Conclusions:**

Our results indicate that PCT determination is a relatively sensitive and specific test for the diagnosis of bacterial peritonitis. However, with regard to methodological limitations and significant heterogeneity, medical decisions should be based on both clinical findings and PCT test results.

**Electronic supplementary material:**

The online version of this article (doi:10.1186/1471-2334-14-452) contains supplementary material, which is available to authorized users.

## Background

Bacterial peritonitis is an inflammation of the peritoneum by micro-organisms such as Gram negative bacilli. The mortality of peritonitis in the early 1900s was close to 90%. With the introduction of various antibiotics, the mortality continued to decrease slowly [1]. However, it is still a common illness that adversely affects the prognosis, and increases costs to health-care systems worldwide. It frequently occurs in children and adults, and can endanger life, particularly in patients who have decompensated cirrhosis or in patients receiving continuous ambulatory peritoneal dialysis therapy. Previous studies showed an average peritonitis rate of 1 episode per 24.5 patient treatment months for continuous ambulatory peritoneal dialysis [2]. While the prevalence of bacterial peritonitis in cirrhotic patients with ascites admitted to hospital ranges ranging 10% to 30% [3]. It has been proven that delayed diagnosis of peritonitis was an important factor for its high mortality, Consequently, diagnosis of bacterial peritonitis continues to be a major clinical challenge, and an accurate biomarker for the early identification of peritonitis would be of great diagnostic value.

Several potential biomarkers have been proposed in highly cited studies for their ability to diagnose bacterial infections, procalcitonin (PCT), which is a precursor of calcitonin including 116-aminoacid polypeptide has been indicated the “the champion” so far [4]. It is undetectable (<0.01 ng/ml) in normal conditions, while in cases of infection, it increases rapidly produced by extrathyroidal cells (e.g. monocytes) [5]. There is a significant body of clinical research indicating a good diagnostic accuracy for the PCT test for discrimination between invasive fungal infection and bacterial infection or noninfectious conditions [4, 6]. However, only one systematic review has investigated the accuracy of PCT for the diagnosis of spontaneous bacterial peritonitis [7], with limitation of the small number of trials included. Additionally, several new studies of PCT have been published and our knowledge of PCT is still developing. Therefore, we undertook the present meta-analysis and systemic review mainly to quantitatively summarize the current evidence on the value of PCT as a marker of bacterial peritonitis. Because there is no consensus about the appropriate PCT cut-off level to predict bacterial peritonitis, and as different PCT thresholds have been used between studies, we calculated the summary receiver operating characteristic (ROC) curves approach to perform this analysis [8].

## Methods

### Data sources and search strategy

**Figure 1:**
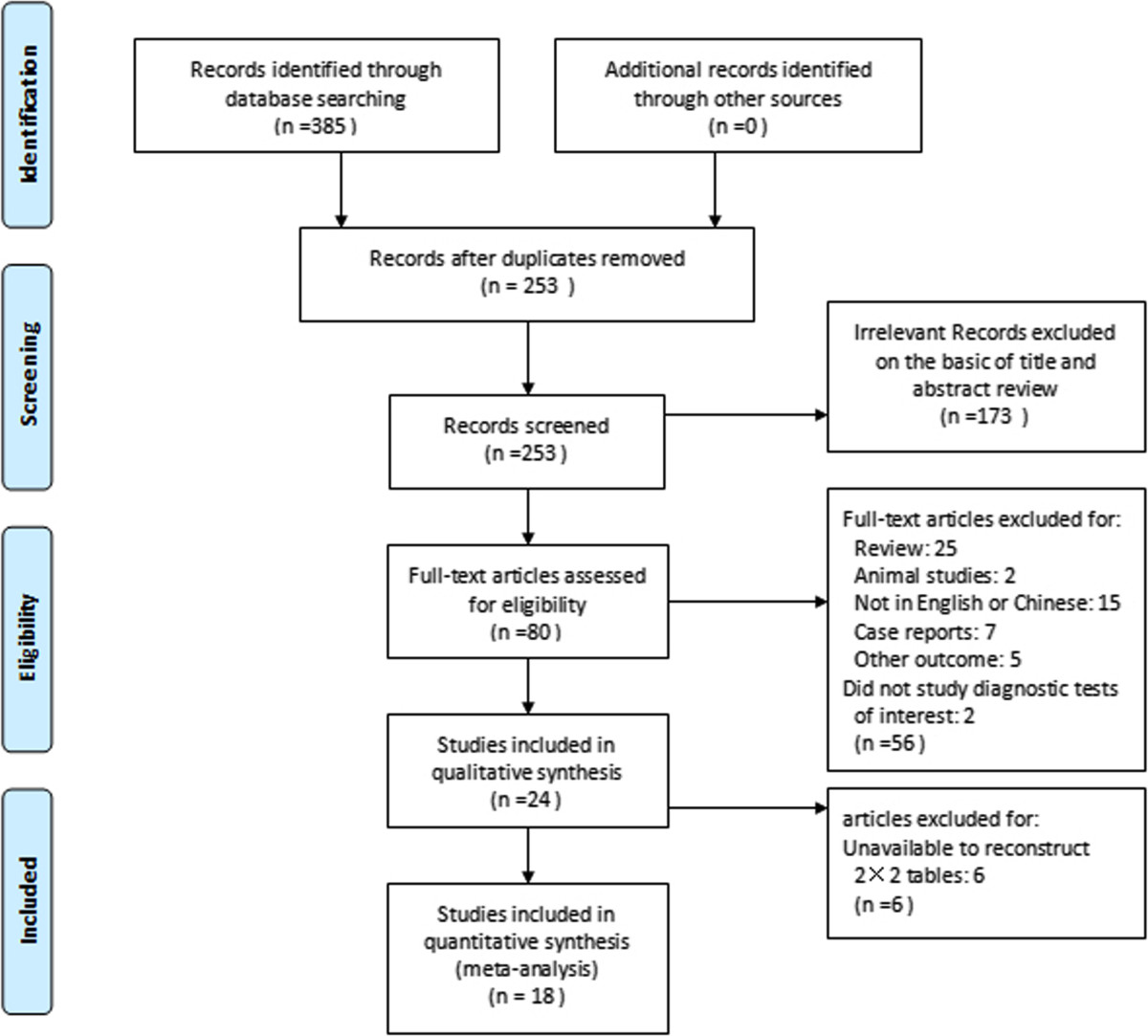
**Flow diagram of study identification and inclusion.**

This meta-analysis was performed according to the meta-analysis of observational studies in epidemiology reporting guidelines [9]. We performed a literature search in MEDLINE, EMBASE, SCOPUS, Cochrane databases, China Biology Medicine Database (CBM), and China National Knowledge Infrastructure (CNKI) Database (all to April 2014) adhering to PRISMA guidelines to identify eligible studies. The following search terms were used: procalcitonin, PCT, peritonitis, ascites, peritoneal, abdominal cavity, infection in combination with biomarker (Figure 1 shows details of the search method used in this meta-analysis). Published studies were sought initially without language restrictions. The patients with any active infection in other organs or sites, active immune disease, or cancer were excluded. We also reviewed the reference lists of the original and review articles in order to identify other potentially relevant trials.

### Study selection

Only studies that investigated the diagnostic accuracy of PCT level to predict peritonitis in humans were considered for inclusion in this meta-analysis. We included prospective and retrospective studies. For the full-text review and the final analysis, we only included articles published in English and Chinese although there was no language restrictions. We excluded case reports, conference abstracts, review and letters to journal editors. Three of the authors (S.K.Y. L.X and P.A.S.) independently evaluated the titles and abstracts, and excluded all studies that were clearly not relevant with the inclusion criteria, then three authors (S.K.Y., P.A.S. and L.X.) assessed the full-text articles independently. We presented the reasons for study exclusion and progress in Figure 1.

### Data extraction and quality assessment

The following data were extracted or recalculated: year of publication, country of origin, sample size, patients ages, study design, PCT testing systems, specificity and sensitivity. The data of 2 × 2 tables (true-positive, false-positive false-negative and true-negative) were calculated for meta-analysis. These data were extracted independently by two authors (S.K.Y. and P.A.S.), and in cases of missing data, we contacted the corresponding author by e-mails in order to seek the required information. We used the Quality Assessment of Diagnostic Accuracy Studies (QUADAS) criteria to assess the quality of included studies [10]. When different sensitivity or specificity values were reported in a study at different cutoffs, the data with the highest Youden index was used for meta-analysis [11]. Then the data of 2 × 2 tables were entered into Stata12.0 by S.K.Y. and the data entry was checked by X.X.X.

### Data synthesis and statistical analysis

The meta-analyses were performed using Stata,version 12.0 (Stata Corp., College Station, TX, USA) [12], notably with the “midas” and “metandi” commands. We calculated the pooled sensitivity and specificity, diagnostic odds ratio (DORs) and the likelihood ratio et al. based on the bivariate random effect models for meta-analysis of diagnostic test data [13]. In case multiple cut-off points for PCT analysis were provided in a same study, we choose the cut-off giving the maximum overall accuracy. We also constructed the respective summary receiver operating characteristic curves(SROC) and calculated the area under the receiver operating characteristic curve (AUROC), irrespective of different cut-off points used [14]. Heterogeneity was quantitatively assessed using the *I*^2^ statistic (*I*^*2*^ value > 50% means a moderate to high heterogeneity) [15]. Remarkable heterogeneity was explored further by subgroup analysis. All statistical tests were two-sided and statistical significance was defined as P < 0.05. To test for possible publication bias, we constructed effective sample size funnel plots versus the log diagnostic odds ratio and did a regression test of asymmetry (Deek’s test), with P < 0.05 for the slope coefficient indicating significant publication bias [16].

## Results

### Identification of studies

Overall, Our electronic search yielded 385 published studies, of which 305 studies irrelevant to this review were excluded after screening titles and abstracts. Having reviewed the full text of the remaining 80 articles, we then excluded another 62 studies: among them, 25 was review article; 7 was case reports; 2 was animal studies, 7 did not investigate the diagnostic value of serum PCT level or explore other outcomes; and 15 was not English or Chinese in the text; and 6 studies was unable to reconstruct 2 × 2 tables. Finally, 18 eligibility studies were included in the analysis [17–34] [Figure 1].

### Study characteristics

The main characteristics of included studies were summarized in Table 1.

**Table 1:** **Characteristics of included studies**

Author, year, country	Design	Mean age (years) Case/control	Patient group	Case/control	Outcomes definition	Biomarkers tested	Cut-off^#^	Sensitivity, specificity	TP(n)	FP(n)	FN(n)	TN(n)	PCT testing assays (FAS of PCT assays)
Cekin, 2013 [21]	Retrospective	63.4	Cirrhotic	59/24	SBP	Serum PCT	0.42	78%, 75%	46	6	13	18	ECLIA (0.5 ng/ml)
Turkey													
Connert, 2003 [25]	Prospective	57	Cirrhotic	19/81	SBP	Serum PCT	0.615	94.7%, 70.4%	18	24	1	57	LUMI test (0.07 ng/ml)
Germany													
Guz, 2006 [17],	Prospective	46.6	PD	21*/35	PDRP	Serum PCT	0.5	62%, 94%	13	2	8	33	LUMI test (0.02 ng/ml)
Turkey						Serum PCT	0.75	43%, 98%	9	1	12	34	
						Serum PCT	1.5	38%, 99%	8	0	13	35	
						Serum CRP	6	95%, 67%	20	12	1	23	
Lai, 2013 [27]	Retrospective	58.2/55.9	Cirrhotic	45/45	SBP	Serum PCT	0.5	83%, 81%	37	9	8	36	ECLIA (0.5 ng/ml)
China						Serum CRP	10	75%, 63%	34	17	11	28	
Lam, 2008 [18],	Prospective	63.8	PD	35/165	PDRP	Serum PCT	0.5	80%, 92%	28	13	7	152	LUMI test (0.06 ng/ml)
Hong Kong													
Liu, 2006 [31]	Retrospective	52/42	Cirrhotic	17/20	SBP	Serum PCT	0.5	100%, 100%	17	0	0	20	Semi-quantitative PCT-Q (0.5 ng/ml)
China													
Liu, 2012 [32]	Retrospective	45.8/43.8	Cirrhotic	55/45	SBP	Serum PCT	0.5	91%, 100%	50	0	5	45	Semi-quantitative PCT-Q (0.5 ng/ml)
China													
Öztürk, 2010 [20]	Retrospective	49.0/44.8	PD	50/50	PDRP	Serum PCT	0.5	42%, 84%	21	8	29	42	Semi-quantitative PCT-Q (0.5 ng/ml)
Turkey						Serum PCT	2	14%, 100%	7	0	43	50	
						Serum CRP	50	40%, 100%	20	0	30	50	
						Serum CRP	8	90%, 11.9%	45	44	5	6	
Spahr, 2001 [22],	Prospective	58.1/57.9	Cirrhotic	10/10	SBP	Serum PCT	0.615	50%, 90%	5	1	5	9	LUMI test (0.07 ng/ml)
Switzerland						Ascitic PCT	0.5	30%, 100%	3	0	7	10	
Viallon, 2000 [23]	Prospective	58.8/57.6	Cirrhotic	21/40	SBP	Serum PCT	0.76	95%, 98%	20	1	1	39	LUMI test (0.07 ng/ml)
France						Serum CRP	80	62%, 92%	13	3	8	37	
						Ascitic PCT	0.3	95%, 85%	20	6	1	34	
Wu, 2014 [33]	Retrospective	50.8/53.0	ESLD	178/184	SBP	Serum PCT	0.443	84.3%, 92.7%	150	14	28	170	Immunoluminometric assay (0.1 ng/ml)
China						Serum PCT	0.462	83.7%, 94.9%	149	10	29	174	
						Serum PCT	0.500	81.0%, 96.1%	144	7	34	177	
Xie, 2014 [34]	Retrospective	28-71	Cirrhotic	56/36	SBP	Serum PCT	0.5	89.3%, 94.4%	50	2	6	34	ECLIA (0.5 ng/ml)
China						Ascitic PCT	0.5	71.4%, 100%	40	0	16	36	
Yang, 2007 [28]	Retrospective	42.4/43.4	Cirrhotic	97/86	SBP	Ascitic PCT	10	92.8%, 96.5%	90	3	7	83	Immunoluminometric assay (0.1 ng/ml)
China													
Yilmaz, 2007 [19]	Retrospective	51.3/50.7	PD	20/20	PDRP	Serum PCT	0.5	70%, 100%	14	0	6	20	Semi-quantitative PCT-Q (0.5 ng/ml)
Turkey						Serum CRP	8	100%, 55%	20	9	0	11	
Yuan, 2013 [24]	Retrospective	55.9/54.8	CSHB	42/42	SBP	Serum PCT	0.48	95%, 79%	40	9	2	33	Automated Immunoanalysis (0.04 ng/ml)
China						Serum PCT	0.67	36%, 98%	15	1	27	41	
						Serum CRP	11.6	86%, 69%	36	13	6	29	
						Serum CRP	16.1	64%, 95%	27	2	15	40	
Zhang, 2004 [29]	Retrospective	51.4	Cirrhotic	41/21	SBP	Serum PCT	13.7	87.8%, 100%	36	0	5	21	Immunoluminometric assay (0.1 ng/ml)
China						Ascitic PCT	9.5	70.7%, 80.9%	29	4	12	17	
Zhang, 2010 [30]	Retrospective	58.0	Cirrhotic	34/39	SBP	Serum PCT	2	79.4%, 89.7%	27	4	7	35	Semi-quantitative PCT-Q (0.5 ng/ml)
China						Serum CRP	8	55.2%, 86.7%	19	5	15	34	
Zhang, 2003 [26]	Retrospective	48.6	Cirrhotic	38/51	SBP	Serum PCT	10	84.2%, 94.1%	32	3	6	48	Semi-quantitative PCT-Q (0.5 ng/ml)
China													

Note: *PD* peritoneal dialysis, *PDRP* peritoneal dialysis-related peritonitis, *FAS* functional assay sensitivities, *PCT* Procalcitonin, *CRP* C-reactive protein, *SBP* Spontaneous bacterial peritonitis, *ECLIA* electrochemiluminescence immunoassay, *CSHB* Chronic severe hepatitis, *ESLD* end-stage liver disease.

^#^PCT, ng/ml; CRP, mg/l, *16 PD patients had 21 episodes of PD peritonitis during the study period.

Nine studies were published in English [17–25], and nine in Chinese [26–34], representing an international experience from 5 countries. There were two trials performed by the same research unit [26, 29], but the patients in these two studies were recruited from different cohorts. Overall, 1827 patients were enrolled. All studies were conducted in adult patients. 4 of them referred to peritonitis in peritoneal dialysis patients [17–20], 12 studies reported spontaneous bacterial peritonitis in cirrhotic patients [21–23, 25–32, 34], 1 study were from spontaneous bacterial peritonitis of chronic severe hepatitis patients [24], and 1 study was from spontaneous bacterial peritonitis of end stage liver disease patients [33]. Fourteen studies [21–34] used ascitic polymorphonuclear(PMN) cells >250/mm^3^, while four studies [17–20] used ascitic PMN >50/mm^3^ as the reference diagnostic standard for peritonitis. Common bacteria isolated were Escherichia coli, and Streptococcus species. PCT levels were measured in serum sample in 17 studies [17–27, 29–34] and in ascites fluid sample in 5 studies [22, 23, 28, 29, 34], mostly using the LUMI test kit (BRAHMS, Berlin, Germany) and Semi-quantitative PCT-Q testing assays.

### Quality assessment

**Figure 2:**
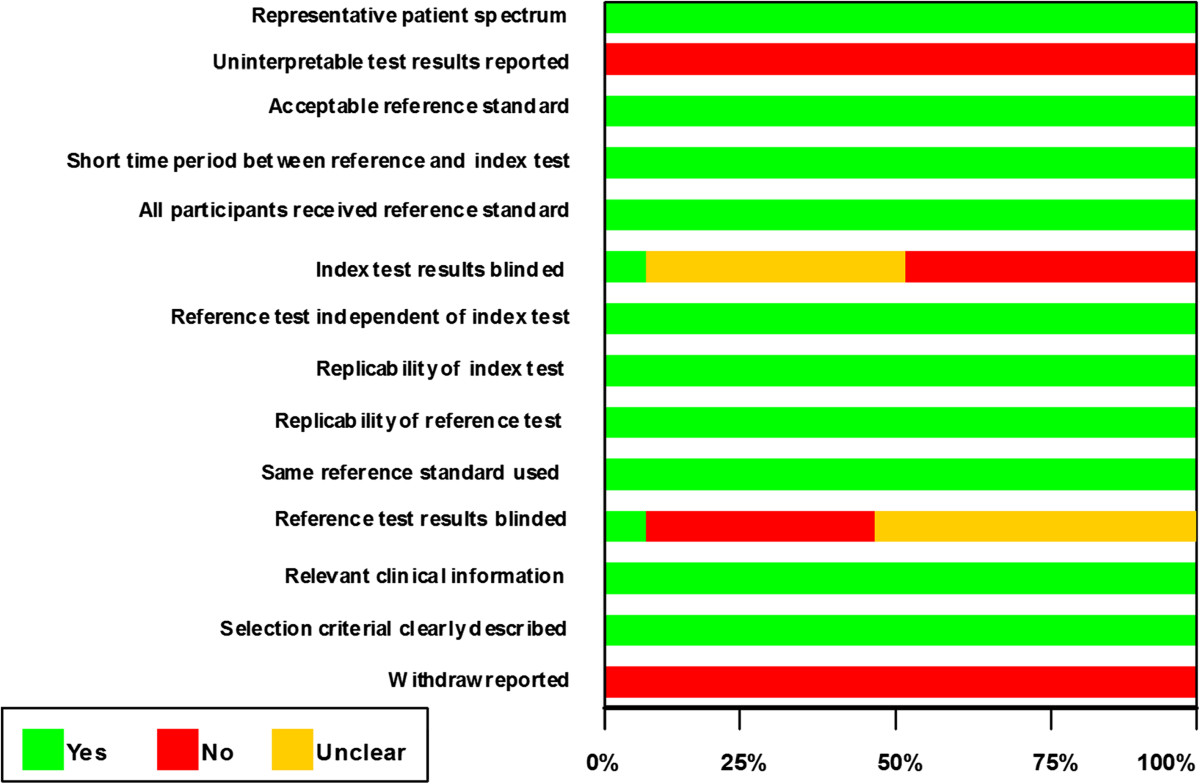
**QUADAS (Quality Assessment of Diagnostic Accuracy Studies) criteria for included studies.**

The QUADAS tool was used for study quality assessment, and Figure 2 provided an overall impression of the quality of the included studies. Five of 18 included studies were prospective [17, 18, 22, 23, 25], and 13 studies were retrospective in design [19–21, 24, 26–34]. All studies had clearly defined inclusion and exclusion criteria. All of these studies used ascitic polymorphonuclear (PMN) cells count, however, as different definitions were used in different trials, which might lead to potential spectrum bias existed.

None of included trials reported withdrawals or uninterpretable result. Eleven studies did not state whether the PCT results were interpreted without knowledge of outcome assessment [17, 18, 21, 24, 28–34], it was poorly reported whether the reference standard results were interpreted blindly, and in only one study, the researchers were blinded to the index test while verifying results by reference standard [23]. These issues might lead to overstated measures of diagnostic accuracy, which is known as a review bias.

### Data extraction and calculation

We calculated the numbers of true-positive, false-positive, false-negative and true-negative based on the provided indexes of sensitivity, specificity, and sample size values. PCT measurement was performed at the beginning of the trial in most of the included studies. We reported the PCT and C-reactive protein (CRP) cut-off values in Table 1. Cut-off values for serum or ascitic PCT varied between studies, ranging from 0.42-13.7 ng/ml, or 0.3-10 ng/ml, respectively.

### Diagnostic accuracy indices

**Figure 3:**
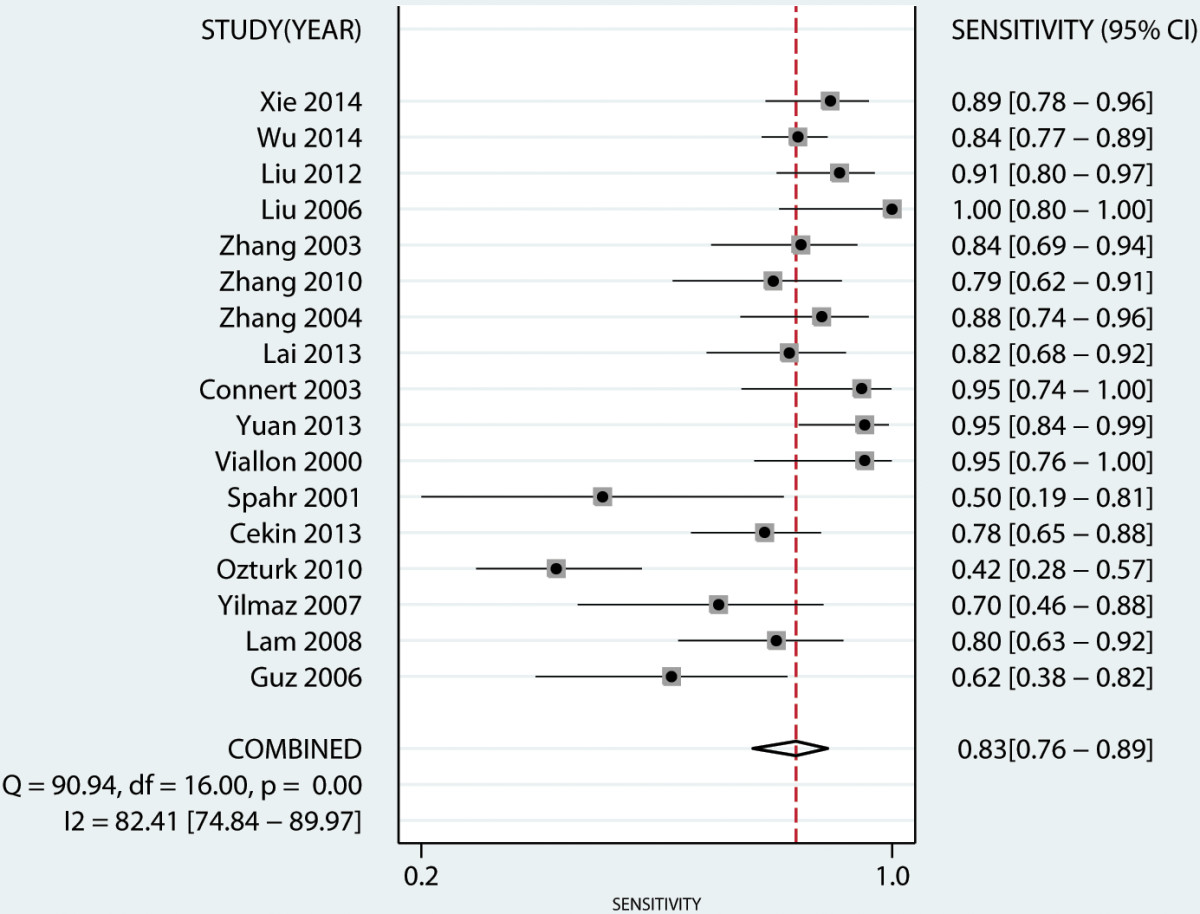
**Forest plot of the pooled sensitivity of serum PCT level in predicting bacterial peritonitis across all settings.**

**Figure 4:**
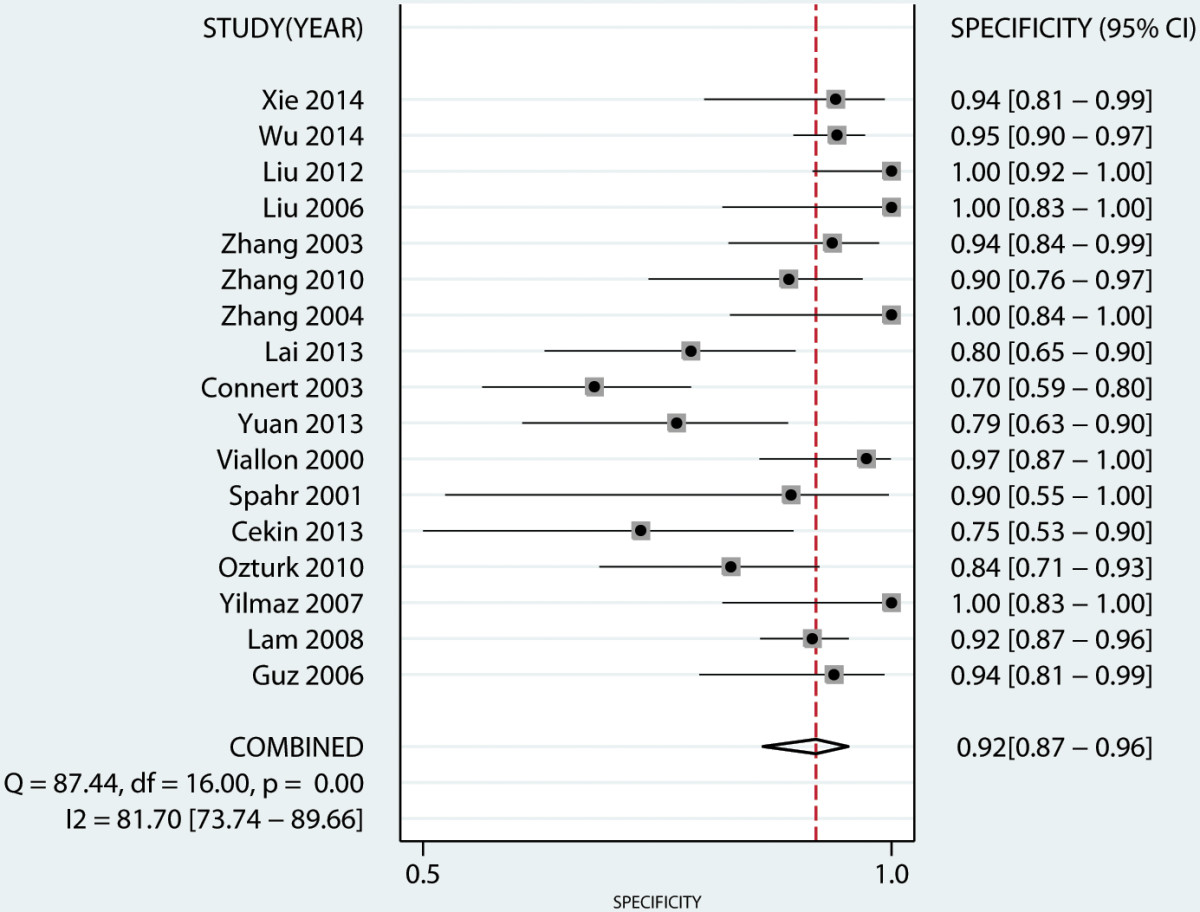
**Forest plot of the pooled specificity of serum PCT level in predicting bacterial peritonitis across all settings.**

**Figure 5:**
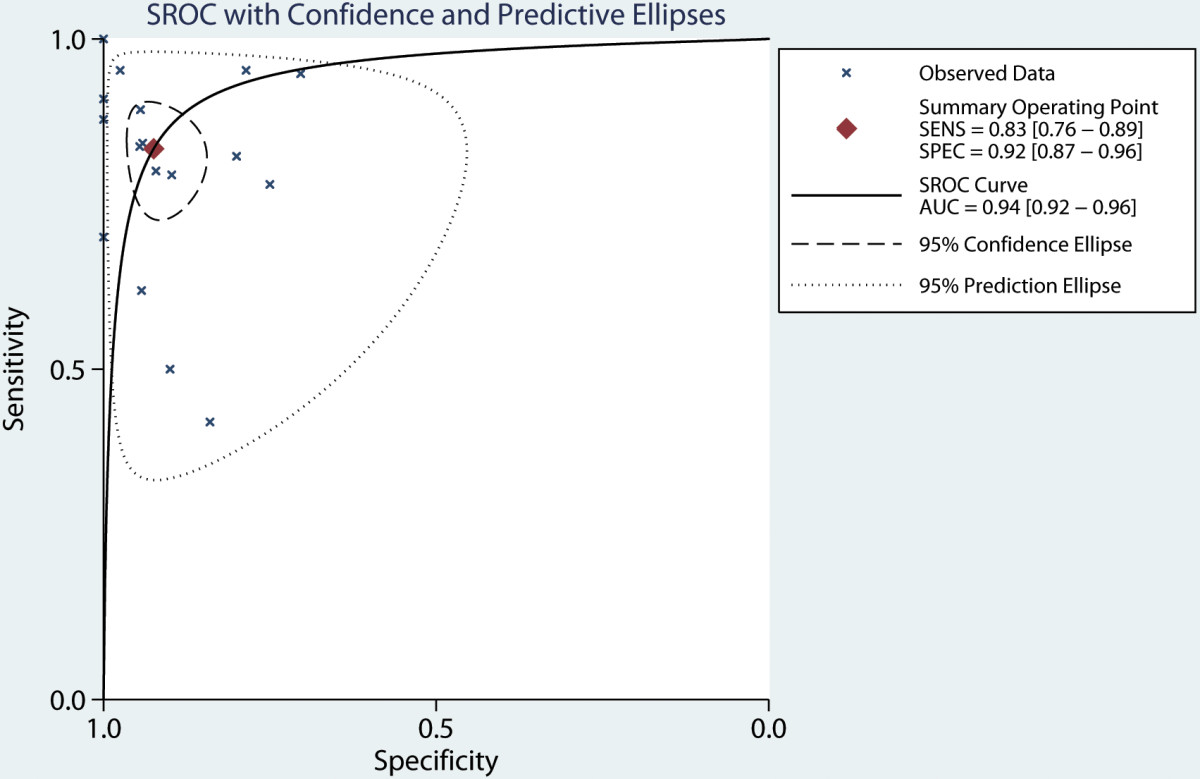
**Hierarchical summary receiver operating characteristic plot of serum PCT level to predict bacterial peritonitis across all settings.** The curve is represented by the straight line, each of the analyzed studies is represented by a fork. The point estimate to which summary sensitivity and specificity correspond is represented by the diamond shape, and the respective 95% confidence intervals are represented by the dashed line. Abbreviation: AUC, area under the curve; SENS: summary sensitivity; SPEC: summary specificity. SROC: summary receiver operating characteristic.

A total of seventeen studies [17–27, 29–34] have investigated the diagnostic value of PCT in serum. Our analysis indicated that serum PCT has a high degree of accuracy for the diagnosis of peritonitis. Pooled sensitivity and specificity estimates of PCT were 0.83 (95% CI: 0.76-0.89), 0.92 (95% CI: 0.87-0.96), respectively (Figures 3 and 4). We also constructed summary ROCs for both PCT and CRP, the results showed that AUROC of PCT was 0.94 (95% CI: 0.92-0.96) (Figure 5). The high positive likelihood ratio (LR+: 11.06; 95% CI: 6.31–19.38) indicates that the PCT test is suitable for a rule-in diagnosis, but its poor negative likelihood ratio (LR−: 0.18, 95% CI: 0.12–0.27) makes it less useful as a rule-out tool.

**Figure 6:**
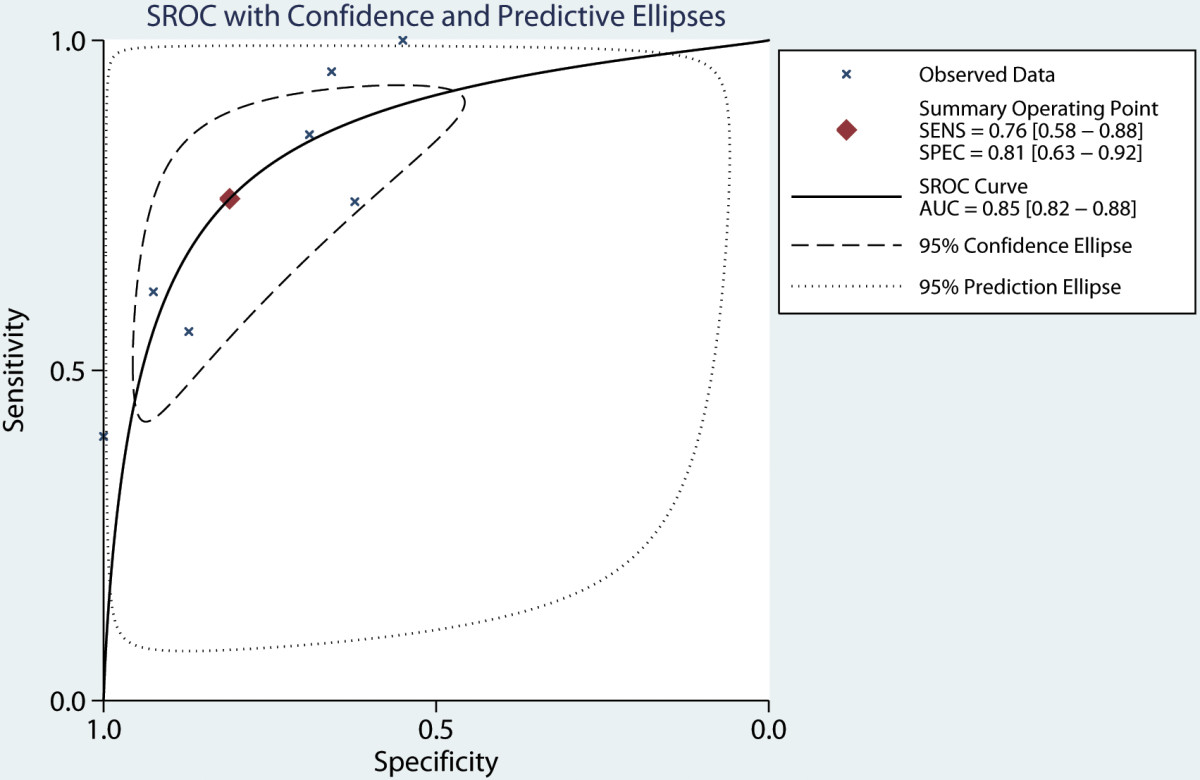
**Hierarchical summary receiver operating characteristic plot of serum CRP level to predict bacterial peritonitis across all settings.** Abbreviation: AUC, area under the curve; SENS: summary sensitivity; SPEC: summary specificity. SROC: summary receiver operating characteristic.

As shown in Table 2, seven studies reported diagnostic value of serum CRP levels [17, 19, 20, 23, 24, 27, 30]. The pooled sensitivity for CRP was 0.76 (95% CI: 0.58–0.88), lower than PCT, the specificity was 0.81 (95% CI: 0.63–0.92), lower than PCT, and the AUROC was 0.85 (95% CI: 0.82-0.88) (Figure 6). CRP has a poorer LR + (4.01, 95% CI: 2.16–7.45) and LR − (0.29, 95% CI: 0.17–0.49). Overall, PCT has a higher discriminative capability than CRP in diagnosing peritonitis. The diagnostic OR for PCT was 61.52 (95% CI: 27.58-137.21), while the diagnostic OR for CRP was 13.54 (95% CI: 7.25-25.28). There was substantial degree of heterogeneity for PCT (*I*^*2*^ = 90.08, 95% CI: 80.35-99.81) and CRP (*I*^*2*^ = 96.81, 95% CI: 94.54-99.09).

**Table 2:** **Summary of subgroup analysis of the included studies by different study characteristics**

Variables	No. of studies	No. of patients	Sensitivity (95% CI)	Specificity (95% CI)	Diagnostic OR (95% CI)	AUROC (95% CI)	***I*** ^2^(95% CI)	Likelihood ratio (95% CI)		Publication bias (Deek’s test P)
								Positive	Negative	
Serum PCT										
Overall analysis	17	1644	0.83(0.76-0.89)	0.92(0.87-0.96)	61.52(27.58-137.21)	0.94(0.92-0.96)	90.08(80.35-99.81)	11.06(6.31-19.38)	0.18(0.12-0.27)	0.973
PD patients	4	391	0.64(0.47-0.78)	0.91(0.85-0.94)	18.33(6.32-53.12)	0.89(0.87-0.92)	0.00(0.00-100.00)	7.19(3.75-13.82)	0.39(0.24-0.62)	0.629
Cirrhotic patients	11	807	0.86(0.81-0.89)	0.94(0.85-0.97)	85.89(27.31-270.19)	0.91(0.89-0.94)	80.65(58.51-100.00)	13.17(5.27-32.86)	0.15(0.11-0.21)	0.734
High cut-off PCT value^a^	9	640	0.68(0.44-0.85)	0.97(0.91-0.99)	60.01(24.19-148.85)	0.95(0.93-0.97)	97.33(95.53-99.13)	20.02(8.29-48.37)	0.33(0.17-0.64)	0.813
Low cut-off PCT value^b^	3	529	0.85(0.80-0.88)	0.89(0.79-0.94)	42.57(19.72-91.89)	0.86(0.83-0.89)	61.42(13.01-100.00)	7.42(3.89-14.17)	0.17(0.13-0.23)	0.299
Common PCT cut-off value^c^	9	1072	0.81(0.68-0.89)	0.95(0.89-0.98)	75.32(21.13-268.52)	0.95(0.93-0.97)	15.30(0.00-100.00)	15.35(6.67-35.28)	0.20(0.12-0.35)	0.973
Common PCT cut-off in PD	4	391	0.64(0.47-0.78)	0.91(0.85-0.94)	18.33(6.32-53.12)	0.89(0.87-0.92)	0.00(0.00-100.00)	7.19(3.75-13.82)	0.39(0.24-0.62)	0.629
Common PCT cut-off in SBP	5	681	0.87(0.78-0.92)	0.97(0.85-0.99)	245.08(26.73-2247.33)	0.94(0.92-0.96)	60.35(10.58-100.00)	33.02(5.06-215.47)	0.13(0.07-0.23)	0.562
Publication in Chinese	8	905	0.86(0.81-0.89)	0.95(0.89-0.98)	141.09(42.44-469.04)	0.93(0.90-0.95)	30.79(0.00-100.00)	20.12(7.64-52.94)	0.14(0.10-0.19)	0.493
Publication in English	9	739	0.79(0.63-0.89)	0.89(0.81-0.94)	29.16(11.78-72.17)	0.92(0.89-0.94)	91.95(84.48-99.43)	6.99(3.97-12.28)	0.24(0.13-0.44)	0.683
FAS > 0.1 ng/ml	9	704	0.82(0.71-0.90)	0.93(0.85-0.97)	65.43(17.30-247.48)	0.95(0.92-0.96)	48.24(0.00-100.00)	12.26(5.01-29.99)	0.18(0.10-0.33)	0.508
FAS ≤ 0.1 ng/ml	8	940	0.86(0.76-0.92)	0.92(0.85-0.96)	71.41(43.93-116.09)	0.95(0.93-0.97)	89.60(79.27-99.92)	10.76(6.01-19.26)	0.15(0.08-0.26)	0.786
Ascitic PCT										
Overall analysis	5	418	0.79(0.54-0.92)	0.96(0.81-0.99)	80.93(15.26-429.28)	0.96(0.94-0.97)	77.94(52.08-100.00)	17.85(4.11-77.59)	0.22(0.09-0.54)	0.075
Serum CRP										
Overall analysis	7	499	0.76(0.58-0.88)	0.81(0.63-0.92)	13.54(7.25-25.28)	0.85(0.82-0.88)	96.81(94.54-99.09)	4.01(2.16-7.45)	0.29(0.17-0.49)	0.676

Note: *PCT* procalcitonin, *PD* peritoneal dialysis, *CRP* C-reactive protein, *SBP* Spontaneous bacterial peritonitis, *FAS* functional assay sensitivities, *AUROC* area under the receiver operating characteristic curve.

^a^High cut-off valu: greater than 0.5 ng/ml; ^b^Low cut-off value: lesser than 0.5 ng/ml; ^c^Common PCT Cut-off value: = 0.5 ng/ml.

There were only 5 studies [22, 23, 28, 29, 34] reporting accuracy parameters on ascitic PCT, across all settings. We found that a diagnostic OR was 80.93 (95% CI: 15.26-429.28) for ascitic PCT level to predict peritonitis at sensitivity and specificity of 0.79 (95% CI: 0.54-0.92) and 0.96 (95% CI: 0.81-0.99) respectively. The pooled positive likelihood ratio was 17.85 (95% CI: 4.11-77.59), negative likelihood ratio was 0.22 (95% CI: 0.09-0.54), and the AUROC was 0.96 (95% CI, 0.94-0.97). The *I*^*2*^ statistic was 77.94%, indicating significant heterogeneity across these studies (Table 2).

### Subgroup analysis

Subgroup analyses were performed to explore the source of heterogeneity. Nine studies [17–20, 27, 31–34] reported test results with the use of a common PCT cutoff value (0.5 ng/mL). The pooled sensitivity and specificity were 0.81 (95% CI: 0.68–0.89) and 0.95 (95% CI: 0.89–0.98) respectively. Three studies [21, 24, 33] also reported diagnostic accuracy parameters using a lower serum PCT cut-off value (0.42-0.48 ng/mL), sensitivity increased appreciably (0.85, 95% CI: 0.80–0.88) and the specificity decreased correspondingly (0.89, 95% CI: 0.79–0.94). In contrast, subgroup analysis on parameters using higher cut-off values (0.615–13.7 ng/mL) showed slightly decreased sensitivity, but increased specificity, compared with the overall estimates [17, 20, 22–26, 29, 30].

There were four studies [17–20] carried out on peritoneal dialysis (PD) patients, as shown in Table 2, The value of serum PCT level to predict peritonitis in PD patients (DOR: 18.33; AUROC: 0.89) was substantially lower compared with that in all included patients (DOR: 61.52; AUROC: 0.94). Eleven studies [21–23, 25–27, 29–32, 34] had reported accuracy parameters on serum PCT in cirrhotic patients. The pooled sensitivity and specificity were 0.86 and 0.94 respectively, and the AUROC was 0.91 (95% CI: 0.89-0.94), indicating that serum PCT also had acceptable predictive performance in cirrhotic patients. The subgroup analyses stratified by the different patient populations (PD and Cirrhotic) using common PCT cut-offs (0.5 ng/ml) showed that the diagnostic value among PD patients (DOR: 18.33; AUROC: 0.89) was substantially lower compared with that in cirrhotic patients (DOR: 245.08; AUROC: 0.94) (shown in Table 2).

As all these included studies using different PCT assays with widely varying functional assay sensitivities (FAS) (shown in Table 1), we have performed the subgroup analyses based on different FAS of the PCT assays, but the result showed that there was no significant difference on the diagnostic value of PCT among trials of different PCT assays (DOR: 65.43; AUROC: 0.95 vs DOR: 71.41; AUROC: 0.95; shown in Table 2). While the subgroup analysis restricted to 8 studies [17, 18, 22–25, 29, 33] using the higher sensitive test tool for PCT (FAS ≤ 0.1 ng/ml) showed the sensitivity increased, but the specificity decreased slightly.

We also performed the subgroup analyses based on different dominated language, and the result showed that the value of serum PCT to diagnosis peritonitis in eight trials published in Chinese (DOR: 141.09; AUROC: 0.93) [26, 27, 29–34] was substantially higher compared with that published in English (DOR: 29.16; AUROC: 0.92; shown in Table 2) [17–25].

### PCT and long-term adverse outcomes of peritonitis

Only two studies reported the relationship between long-term survival outcome and the PCT level. Lam et al. [18] found that there was no difference in the survival rate between PD patients with PCT >0.38 ng/mL and patients with PCT <0.38 ng/mL (p = 0.37). However, Connert et al. [25] reported that cirrhotic patients with PCT levels above 0.58 ng/ml were associated with poor survival compared to those with levels below 0.58 ng/ml, and significant differences were found in mean PCT level between patients who died or survivied during the follow-up period (p < 0.01).

### Publication bias

We used funnel plots to assess the publication bias, as shown in Table 2, there was no significant evidence of potential publication bias was noted by Deek’s test.

## Discussion

PCT is a polypeptide of 116 amino acids (molecular weight 13 kDa) with a long half-life of 25-30 h [5]. It was first identified during studies of hypocalcaemia associated with Staphylococcal toxic shock syndrome [35], and then in 1993, its elevated level was found in patients with bacterial infection. It is most commonly produced from neuroendocrine cells in non-thyroidal tissues such as lung, liver or kidney during inflammation [36, 37]. Unlike other inflammatory markers (e.g., CRP), PCT level does not respond to viral infection or sterile inflammation. In the serum of healthy individuals, PCT is undetectable (<0.01 ng/ml) and a value of >0.5 ng/ml is considered abnormal [5, 36]. Many studies have shown that PCT could rapidly increase in response to bacterial inflammatory stimuli, and it could be recommended as an effective biomarker in the detection and differential diagnosis of inflammatory states [4, 6, 38, 39]. However, there are few meta-analyses on the accurancy of PCT in predicting bacterial peritonitis. In our analysis, which included 1827 patients, we showed that the measurement of serum PCT could provide considerable predictive value (DOR: 61.52; AUROC: 0.94) for the diagnosis of bacterial peritonitis, and that this predictive capacity is better than that provided by CRP (DOR: 13.54; AUROC: 0.85).

There are many types of bacterial peritonitis. In clinical practice, PD related peritonitis and spontaneous bacterial peritonitis (SBP) in cirrhotic patients are most common types in clinical practice. PD represents an important method for the management of uremic patients. Despite a decreasing incidence of PD-related infectious complications over the last couple of decades, peritonitis remains a leading complication and a common cause of infection-related mortality in PD patients [40, 41]. Microbiological culture systerm in PD effluent is the gold standard for diagnosis of PD-associated peritonitis, but suffers from high false negative rates and delayed reporting [40]. In this regard, the use of some new reliable diagnostic markers such as PCT can greatly improve the turnaround time of laboratory reports. It has been demonstrated that renal elimination is the major pathway for clearance of PCT [42], and a previous study performed by Opatrna et al. [43] showed that the serum levels of PCT were increased in PD patients without overt signs of infection compared with healthy volunteers. But follow-up studies revealed the plasma clearance rate of PCT correlated weakly with renal function dysfunction, and it might not influence clinical decisions based on PCT [42, 44]. Our meta-analysis also confirmed an acceptable diagnostic accuracy for PCT testing in PD patients (Sensitivity, 0.64; Specificity, 0.91; DOR: 18.33; AUROC: 0.89).

SBP is the most frequent and life-threatening infection in decompensated cirrhotic patients [45]. Owing to an inadequately immune response, clinical manifestations of SBP in cirrhotic patients may be atypical. There is considerable evidence indicating that high PCT levels may be related to infections in cirrhosis [25]. Although the liver is considered as the main source of PCT, a study performed by Bota et al. [46] showed that serum levels of PCT did not significantly decrease in cirrhotic patients. Moreover, PCT had similar predictive power for infection in patients with and without cirrhosis. In accordance with these findings, our meta-analysis also showed that serum PCT testing has a good accuracy for the diagnosis of bacterial peritonitis in cirrhotic patients (Sensitivity, 0.86; Specificity, 0.94; DOR: 85.89; AUROC: 0.91).

Some authors postulated that ascitic PCT might be more sensitive than serum PCT for the early identification of peritonitis, because bacterial infection could trigger peritoneal inflammatory cells to produce PCT, which then may accumulate in the ascitic fluid. In the present study, the pooled analysis of 5 studies [22, 23, 28, 29, 34] suggested that ascitic PCT (DOR: 80.93; AUROC: 0.96) to was similar with serum PCT (DOR: 85.89; AUROC: 0.91) in diagnosing peritonitis in cirrhotic patients. And Viallon et al. found that PCT detection in ascitic fluid was due to hyperpermeability of peritoneum, while PCT was not synthesized by leucocytes in ascites [23]. These small and in part not significant differences rather supported the assumption of Viallon et al. that PCT detection in the ascitic fluid was the result of a passive shift due to increased vascular permeability instead of an intraperitoneal synthesis. Considering the serious harm for the missed diagnoses of peritonitis, it is not recommended to use ascitic PCT testing as a stand-alone test, and more larger prospective trials are needed to fully elucidate the potential diagnostic value of ascitic PCT.

Fungal peritonitis is a quite uncommon but potentially fatal complication both in peritoneal dialysed [47] and advanced liver cirrhosis patients [48]. The clinical characteristics of fungal peritonitis is not typical and easy to be misdiagnosed. In addition, fungi infections usually result in treatment failure with antibacterial agents and even removing the PD catheter. Early recognition of fungal peritonitis allows for timely and effective therapy with improved outcome, but it is hampered by a lack of a reliable diagnostic tool. There is a significant body of clinical research indicates good diagnostic accuracy for the PCT test for discrimination between invasive fungal infection and bacterial infection [6, 49]. However, the differential diagnostic value of the PCT testing on fungal peritonitis has not been explored. It is speculated that the PCT testing can provide effective sensitivity and specificity for distinguishing fungal peritonitis from bacterial peritonitis.

The pooled likelihood ratio estimates (LR^+^ and LR^−^) was analyzed to calculate post-test probabilities. In a virtual population with a 20% prevalence of peritonitis (the actual prevalence of SBP in hospitalized cirrhotic patients with ascites was 10-30% [50]), use of a serum PCT test with an LR + of 11.06 would increase the posttest probability (positive predictive value) to 72%. In other words, about 3 in 4 patients with positive PCT test results may have confirmed peritonitis. Likewise, in the same population, application of a serum PCT test with a negative likelihood ratio of 0.18 would reduce the post-test probability to 5%, In other words, 1 in 20 patients with negative PCT results may have peritonitis. Using data from the subgroup with a higher PCT cut-off value (0.615–13.7 ng/mL), a similar calculation indicated a positive post-test probability of 85% and a negative post-test probability of 7%.

There was substantial heterogeneity detected for the overall results between the eighteen included studies. Potential source of heterogeneity included the different characteristics of the studies, such as methodological quality, admission category, size of the study populations, different reference standards in PD or cirrhotic patients for peritonitis (ascitic PMN > 50/mm^3^ or > 250/mm^3^, respectively), different countries and different human race and different methods used for measurement of PCT (LUMItest, ECLIA and the Semi-quantitative PCT-Q assay systems). And other unrecorded differences among these studies might also contribute to the heterogeneity. Evaluation with individual patient data or meta-regression would help in this analysis of the sources of heterogeneity. However, the meta-regression would have to adjust for factors at individual patient level, which were not available at present, therefore it limited our ability to further evaluate heterogeneity. On the other hand, using more homogeneous trials could solve this difficulty, but it could induce selection bias.

There are several potential limitations to our study that should be addressed.

First, in our meta-analysis, various PCT testing assay tools and various PCT cut-off values were used in different included studies, and sensitivities and specificities varied between studies. We performed subgroup analysis and constructed the HSROC curve and calculated AUROC to diminish the influence of different PCT assays and cut-off values effect. Despite the adjustment by bivariate model, there might be residual influence on the accuracy of pooled diagnostic parameters. Second, most of the studies have a case–control design, it has been demonstrated that the case–control design could over-estimate the accuracy of a diagnostic test [51], therefore more larger prospective trials should be performed to elucidate the diagnostic value of PCT. Third, as mentioned before, our study suffered from moderate heterogeneity, mainly owing to different patients characteristics, and different definitions of peritonitis. Fourth, despite no significant publication bias was detected, however, this study included 9 low-quality Chinese language trials, which might lead to an overestimation of overall diagnostic accuracy of PCT, especially because positive studies were more easily reported.

## Conclusion

In conclusion, our meta-analysis shows that PCT is a helpful marker in identifying bacterial peritonitis, Although PCT performs as well in PD patients as in cirrhotic or severe hepatitis patients, use of a common cut-off value may further enhance accuracy. Compared with CRP, PCT is superior in the diagnosis of bacterial peritonitis. However, it is important to note that PCT cannot be recommended as a “gold standard” test for peritonitis up to now, and should be interpreted in combination with other clinical, analytical, and/or microbiological data. Given the limits of PCT as a single marker, additional large prospective studies should determine its diagnostic value in bacterial peritonitis, when interpreted in association with other biomarkers.

## Authors’ original submitted files for images

Below are the links to the authors’ original submitted files for images. Authors’ original file for figure 1 Authors’ original file for figure 2 Authors’ original file for figure 3 Authors’ original file for figure 4 Authors’ original file for figure 5 Authors’ original file for figure 6
