# Supplementary figures and images for: Significance of serum procalcitonin as biomarker for detection of bacterial peritonitis: a systematic review and meta-analysis
Source: BMC Infect Dis. 2014 Aug 22;14:452. doi: 10.1186/1471-2334-14-452 (PMC4155125; doi:10.1186/1471-2334-14-452)

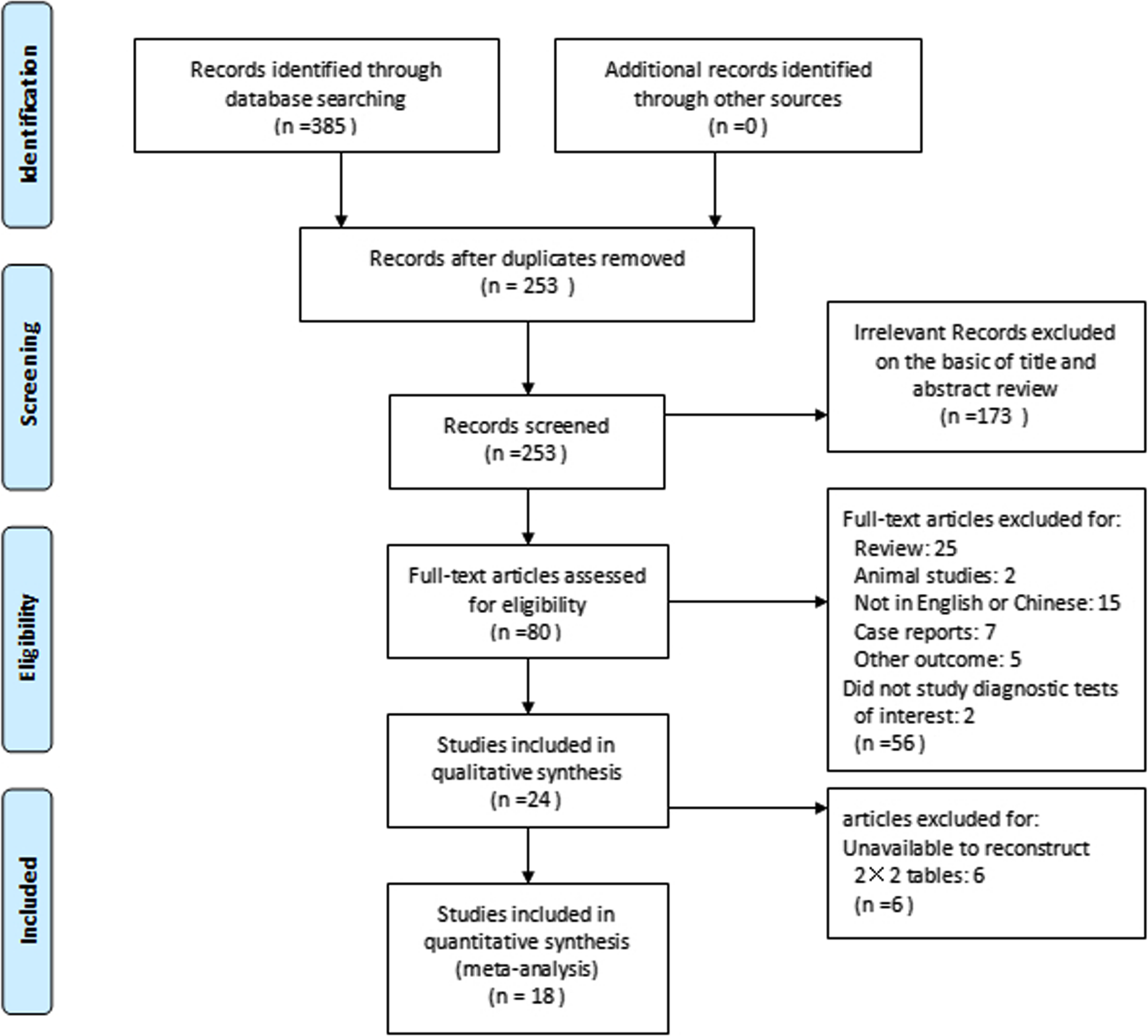

Supplement: Supplementary file 1 — Authors’ original file for figure 1 [file 12879_2013_3758_MOESM1_ESM.tiff]

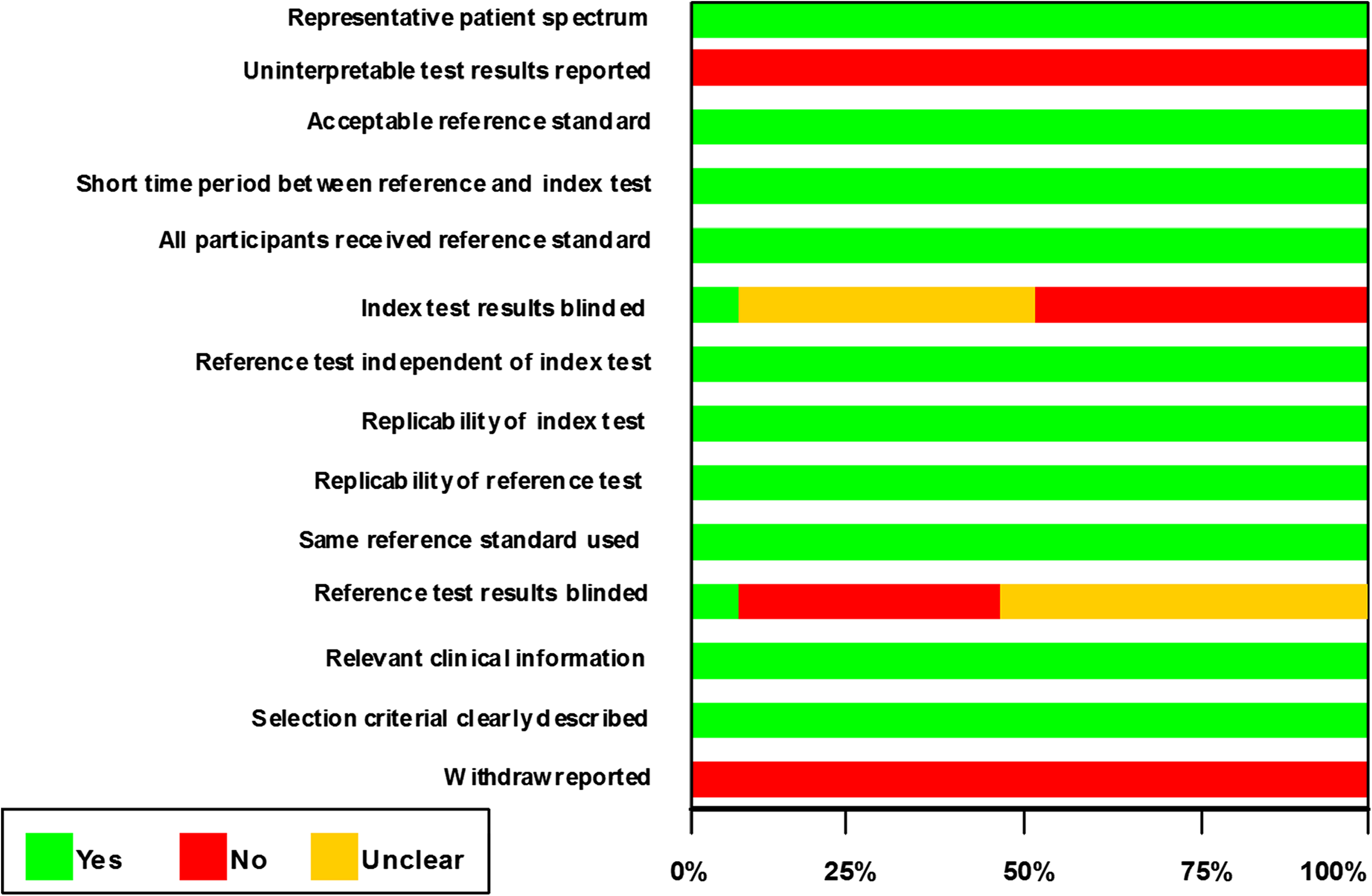

Supplement: Supplementary file 2 — Authors’ original file for figure 2 [file 12879_2013_3758_MOESM2_ESM.tiff]

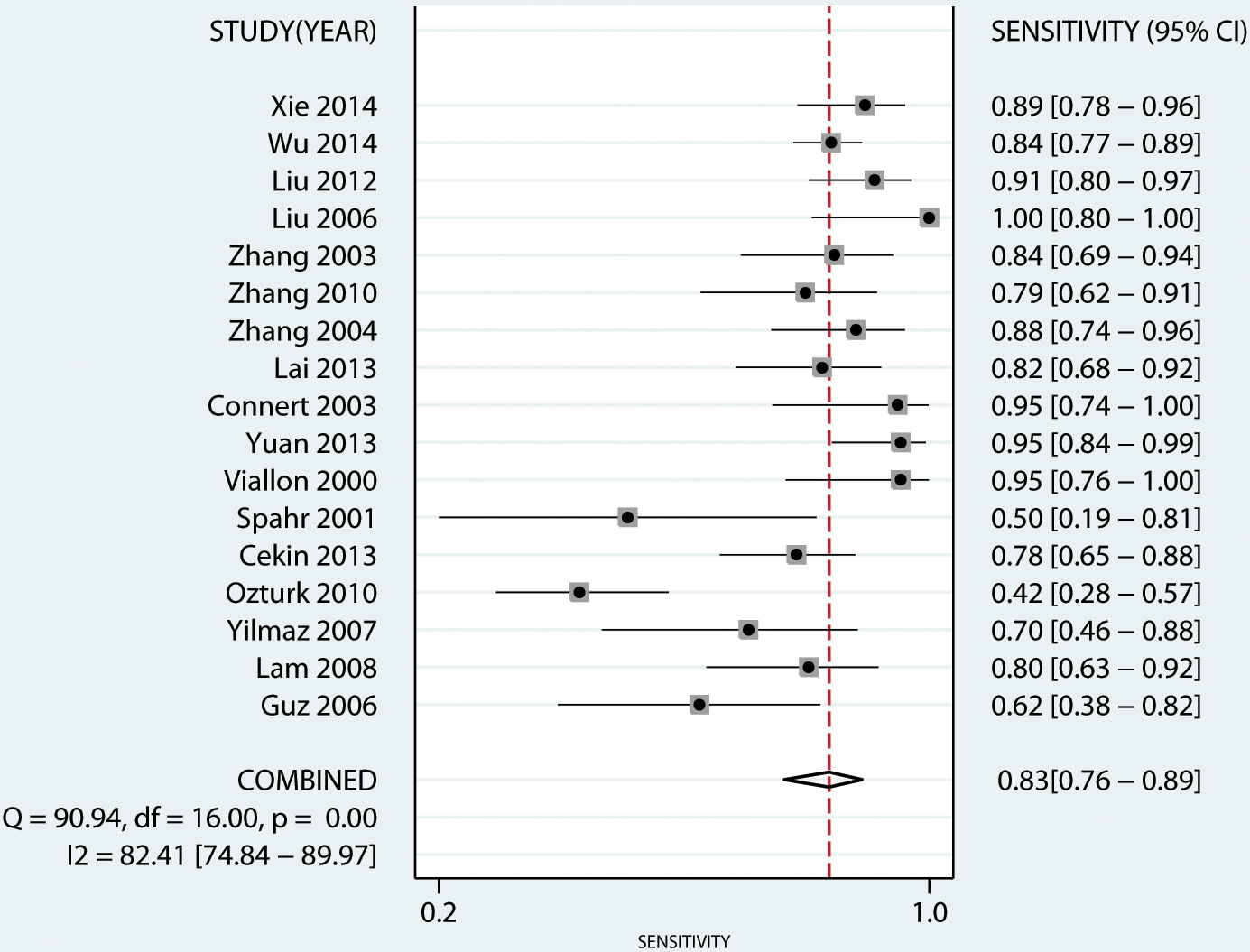

Supplement: Supplementary file 3 — Authors’ original file for figure 3 [file 12879_2013_3758_MOESM3_ESM.tif]

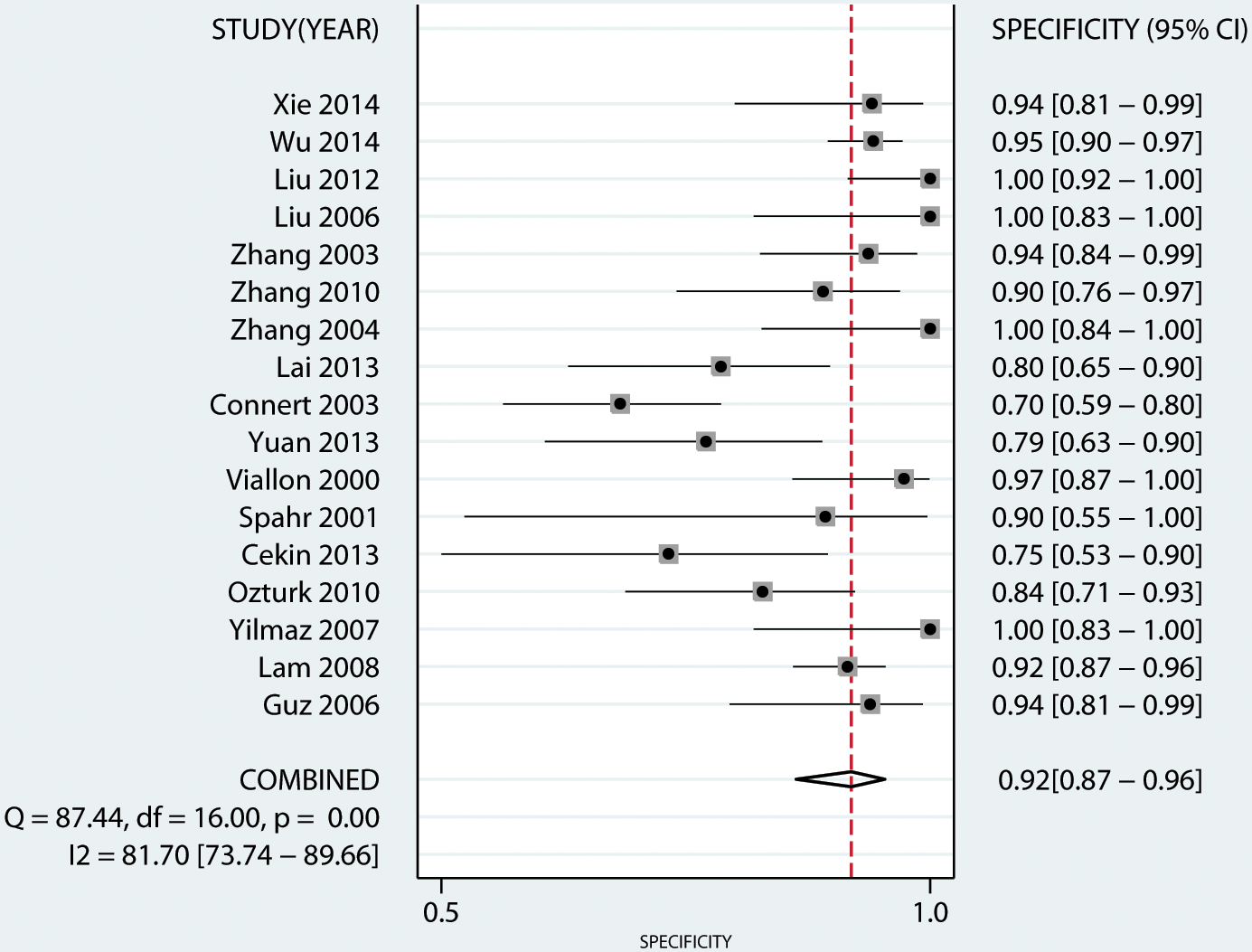

Supplement: Supplementary file 4 — Authors’ original file for figure 4 [file 12879_2013_3758_MOESM4_ESM.tif]

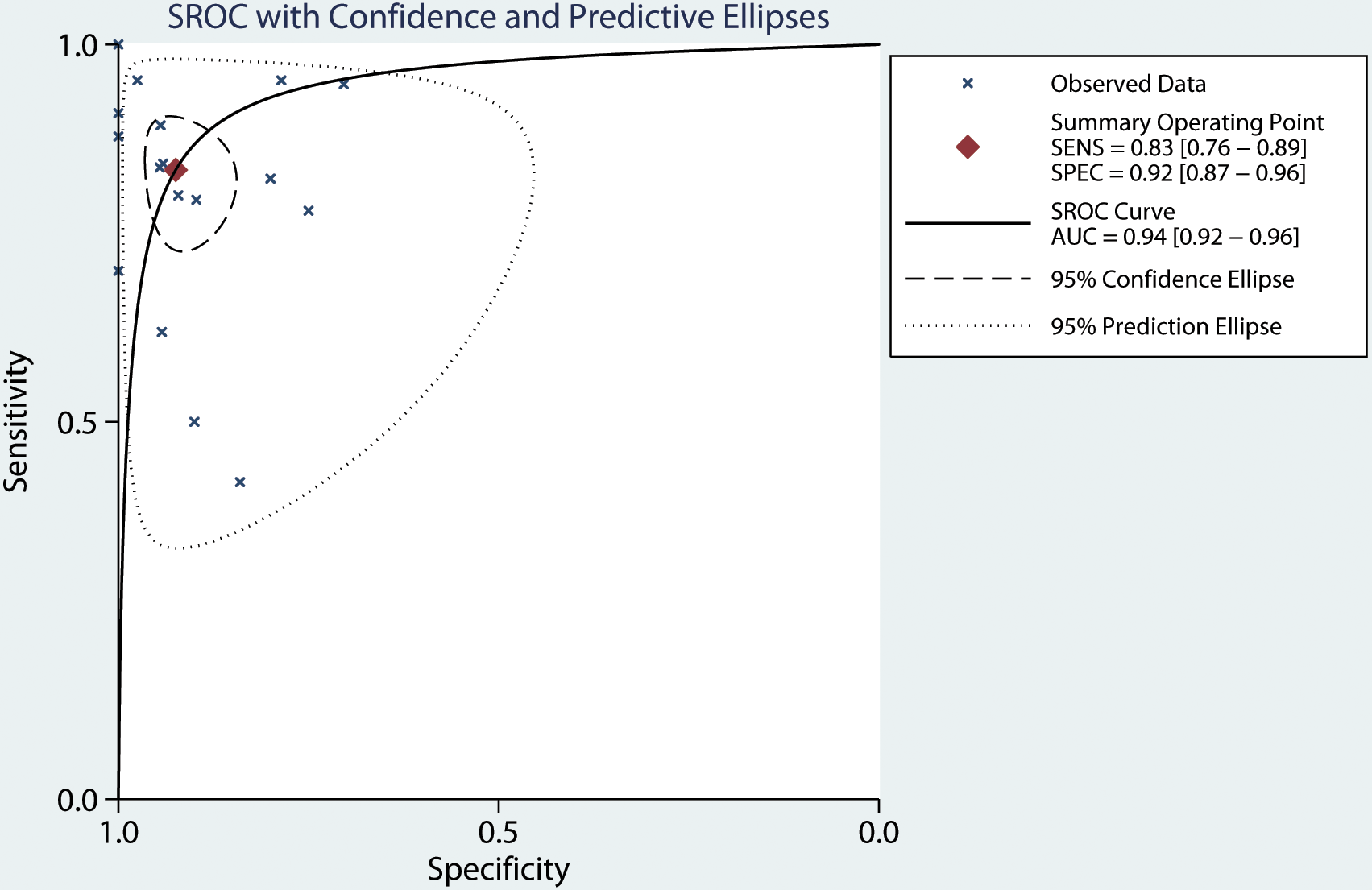

Supplement: Supplementary file 5 — Authors’ original file for figure 5 [file 12879_2013_3758_MOESM5_ESM.tif]

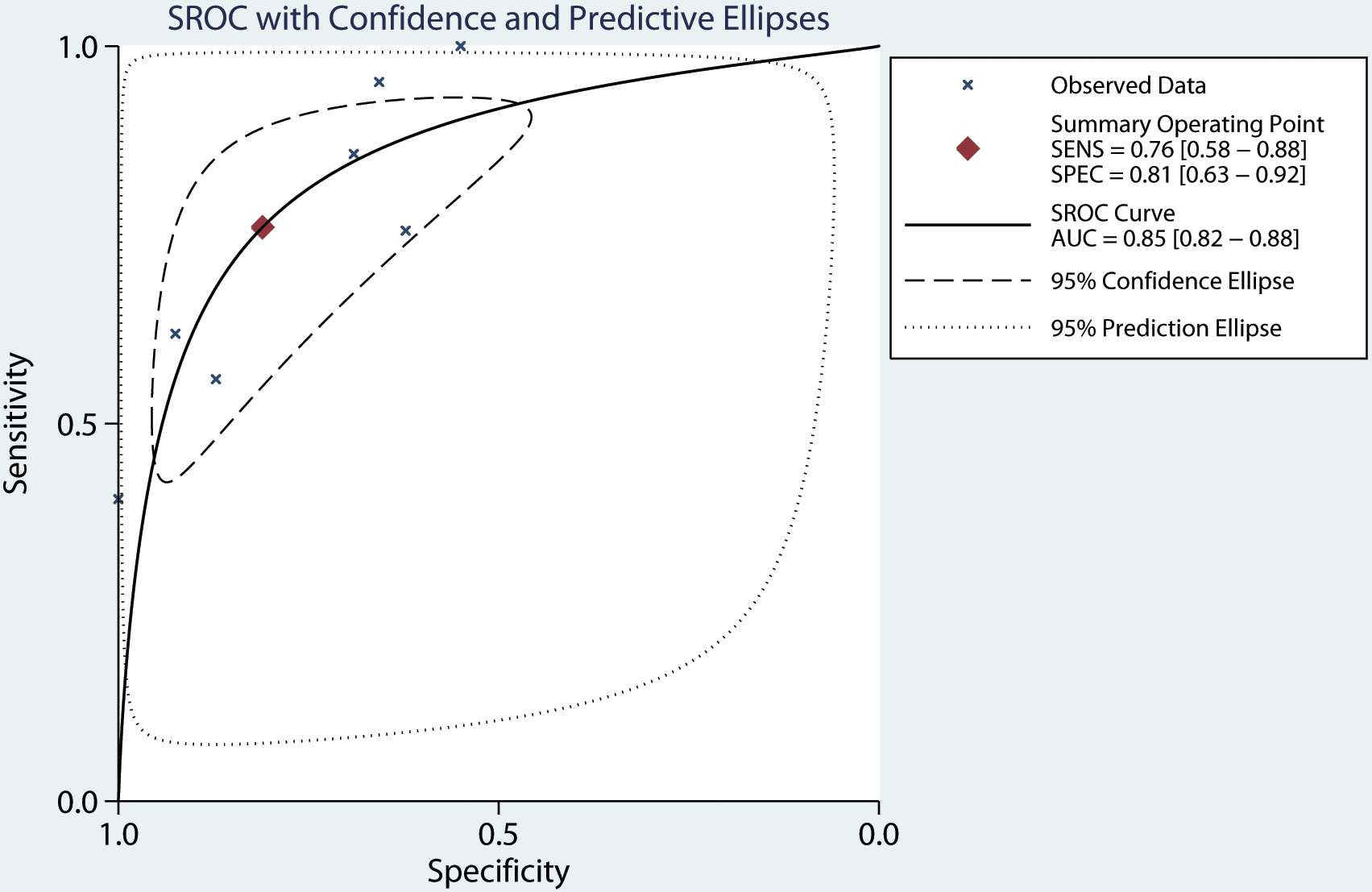

Supplement: Supplementary file 6 — Authors’ original file for figure 6 [file 12879_2013_3758_MOESM6_ESM.tif]
